# Editorial: Advances in molecular plant pathology, plant abiotic and biotic stress

**DOI:** 10.3389/fpls.2024.1398469

**Published:** 2024-03-22

**Authors:** Laura Medina-Puche

**Affiliations:** Department of Plant Biochemistry, Center for Plant Molecular Biology (ZMBP), Eberhard Karls University, Tübingen, Germany

**Keywords:** plant-pathogen interactions, plant viruses, abiotic stress responses, plant pathogenic bacteria, plant immunity, soil-pathogen interactions, disease management strategies, herbicide resistance mechanisms

Frontiers in Plant Science is delighted to present a diverse and comprehensive collection of research articles in the esteemed Research Topic titled “*Advances in Molecular Plant Pathology, Plant Abiotic and Biotic Stress*”. Among the 18 articles featured within this Research Topic, several investigations offer significant contributions to our understanding of molecular plant pathology and plant responses to abiotic and biotic stresses. This compilation serves as a testament to the relentless efforts of researchers worldwide in unraveling the complexities of plant responses to various stressors, thereby advancing our understanding of plant biology, and bolstering agricultural sustainability.

## Abiotic stress responses

Contributions focusing on plant responses to abiotic stresses offer valuable insights into stress perception, signal transduction, and tolerance mechanisms. Tu et al. present a comprehensive review discussing the current advances in understanding the molecular regulation of abiotic stress tolerance in sorghum. This review synthesizes recent progress in physiological, transcriptomic, proteomic, and metabolomic studies, highlighting the molecular mechanisms underlying sorghum’s remarkable tolerance to diverse stressors. Furthermore, it emphasizes the importance of studying combined abiotic stresses, offering valuable insights for enhancing stress tolerance in crops crucial for global food security.

In the work carried out by Guo et al. explore the influence of temperature on glyphosate efficacy on glyphosate-resistant and -susceptible goosegrass (*Eleusine indica*). This study reveals the intricate interplay between temperature and herbicide efficacy, demonstrating that temperature fluctuations significantly affect glyphosate performance on different biotypes of *E. indica*. By elucidating the mechanisms underlying temperature-mediated variations in herbicide efficacy, this research provides valuable insights for glyphosate application and resistance management in weed control practices.

Qiu et al. delve into the adaptation mechanisms of NaHCO_3_-tolerant chlorella and uncover the critical role of cellulose in conferring resistance to carbonate stress. By investigating cell wall polysaccharide composition and gene expression patterns, the researchers unveil the molecular basis of NaHCO3 tolerance in chlorella, highlighting the significance of cell wall-related genes, particularly *JbKOBITO1*, in mediating cellulose accumulation and stress resistance. These findings offer valuable insights into the genetic resources for crop breeding and the genetic modification of microalgae for sustainable biofuel production.

## Advances in plant-pathogen interactions: insights from systems genomics to molecular mechanisms

Several articles within this Research Topic deepen into the molecular mechanisms underlying plant-pathogen interactions, shedding light on strategies employed by both plants and pathogens during infection.

### Deciphering resistance mechanisms across multiple pathosystems

Notable among these is the work by Million et al., which employs a systems genomics approach to elucidate the molecular mechanisms underpinning quantitative resistance to *Phytophthora sojae* in *Glycine max*. Furthermore, Tun et al. elucidate the role of sucrose in promoting defense responses to blast fungus in rice through the preferential expression of defense-related genes.

In the study by Xu et al., the authors investigate the molecular mechanisms underlying iron (Fe)-mediated tobacco resistance to potato virus Y (PVY) infection. This study provides novel insights into the regulatory network of Fe-mediated resistance to PVY infection in plants. By elucidating the transcriptomic responses and identifying key candidate genes involved in PVY resistance, this research offers important theoretical bases for improving host resistance against PVY infection, thus contributing to the development of effective disease management strategies. Another contribution in the realm of plant-pathogen interactions is the study elaborated by Chang et al. which sheds light on the intricate dynamics of virus-virus interactions and their impact on mechanical transmissibility and host range alterations of begomoviruses. Their findings reveal how mixed infections can influence the transmission efficiency and host specificity of these pathogens, providing valuable insights for disease management strategies.

Zhao et al. investigate the responses of sugarcane to two strains of *Xanthomonas albilineans* differing in pathogenicity. By employing molecular analyses, they elucidate the differential modulation of salicylic acid and reactive oxygen species in the sugarcane defense response to pathogen attack. This study provides novel insights into the pathogenic diversity of *X. albilineans* and the molecular mechanisms underlying sugarcane defense strategies. Similarly, Gao et al. conduct a comparative transcriptome profiling of susceptible and tolerant citrus species infected with “*Candidatus liberibacter* asiaticus,” offering novel perspectives on citrus Huanglongbing disease management. Chen et al. characterize gene expression patterns in response to orthotospovirus infection between two diploid peanut species and their hybrid, shedding light on the genetic basis of virus resistance in peanut. In the publication led by Zhao et al., the authors explore genetic and morphological variants of *Acidovorax avenae* subsp. *avenae* causing red stripe disease of sugarcane in China, offering insights into the genetic diversity and virulence mechanisms of this pathogen.

### Understanding plant immunity and stress responses

The Research Topic also encompasses studies addressing a range of other pathogens and stress factors. In the study by Lipps et al., the authors delve into the inhibition of ethylene and its role in resistance to Northern corn leaf blight (NCLB) in maize. By employing a multifaceted approach combining genome-wide association studies, metabolic pathway analyses, and ethylene inhibition experiments, the researchers elucidate the complex interplay between genetic factors, metabolic pathways, and hormonal signaling in maize resistance to NCLB. This study not only identifies novel markers associated with NCLB resistance but also underscores the importance of ethylene in plant defense against fungal pathogens, paving the way for future investigations into the genetic basis of disease resistance. In addition, Yang et al. shed light on the pivotal role of microRNAs (miRNAs) in tobacco defense against *Phytophthora nicotianae*, the causal agent of tobacco black shank. Through meticulous experimentation and genetic manipulation, the researchers demonstrate that overexpression of Nta-miR6155 enhances tobacco resistance to *P. nicotianae* infection by modulating reactive oxygen species (ROS) levels, salicylic acid signaling, and the expression of key defense-related genes. Moreover, the study elucidates the regulatory role of Nta-miR6155 in tobacco growth and development, underscoring the multifaceted functions of species-specific miRNAs in plant stress responses.

The contribution to this Research Topic coming from Shang et al. explore the dynamic interplay between light intensity and soybean mosaic virus (SMV) infection in soybean. Through RNA-seq analysis and gene expression profiling, the researchers uncover the pivotal role of light in modulating soybean defense responses to viral infection. This study enhances our understanding of the molecular mechanisms underlying plant-virus interactions and highlights the importance of environmental factors in shaping plant defense strategies.

An original approach is presented by Li et al.. The authors investigate the potential of exogenous melatonin application in enhancing radish defense against *Alternaria brassicae*, the causative agent of radish blight disease. Through a combination of physiological assays and transcriptomic analyses, the researchers reveal the dose-dependent effects of melatonin on both host immunity and pathogen virulence. Their findings provide a mechanistic understanding of melatonin-mediated defense responses in radish, offering promising prospects for the development of melatonin-based strategies for disease control in vegetable crops.

### Unraveling soil-pathogen interactions

Gauthier et al. contribute to our understanding of soil-borne wheat mosaic virus (SBWMV) and Soil-borne cereal mosaic virus (SBCMV) infection in wheat by investigating the influence of soil properties and plant nutrition on virus infection rates. Through meticulous analyses of soil structure parameters, nutrient contents, and infection rates, the researchers reveal the intricate relationship between soil characteristics, plant nutrition, and virus transmission. These findings not only offer insights into the environmental factors influencing virus infection but also provide potential avenues for developing microenvironment-adapted agricultural practices to mitigate virus spread.

## Investigating alternative disease management strategies and exploring herbicide resistance mechanisms in weed species

Trouvelot et al. investigate the impact of sodium arsenite on grapevine physiology and its potential as an alternative treatment for grapevine trunk diseases (GTDs). By employing metabolomic and histological analyses, the researchers elucidate the effects of sodium arsenite on wood tissues and secondary metabolite production, shedding light on its mode of action against GTD pathogens. This study contributes valuable insights into the development of sustainable strategies for GTD management, addressing the urgent need for eco-friendly alternatives to conventional treatments.

Cao et al. provide a comprehensive analysis of metamifop resistance in *Digitaria ciliaris* var. *chrysoblephara*, a problematic grass weed in China. Through genome sequencing, gene expression profiling, and whole-plant bioassays, the researchers unravel the molecular mechanisms underlying metamifop resistance, identifying a target-site mutation in the ACCase gene associated with resistance. Additionally, the study elucidates cross-resistance patterns to other herbicides, offering valuable insights into the management of herbicide-resistant weed populations.

## Future directions and implications/concluding remarks

As global climate change escalates, understanding plant stress responses becomes increasingly critical. By deciphering the molecular basis of these responses, researchers not only empower farmers with tools to mitigate crop losses but also contribute to the sustainable intensification of agriculture, promoting both environmental stewardship and economic viability.

Collectively, these articles underscore the interdisciplinary nature of research in plant science and highlight the interconnectedness of molecular mechanisms governing plant responses to diverse stressors. By elucidating these mechanisms, researchers pave the way for the development of resilient crops capable of withstanding environmental challenges, thereby ensuring global food security and sustainability.

## Author contributions

LM-P: Conceptualization, Writing – original draft, Writing – review & editing.

